# Macro Pituitary Adenoma and Frontal Calcified Cavernous Malformation: A Coincidence or a True Partnership?

**DOI:** 10.7759/cureus.21152

**Published:** 2022-01-12

**Authors:** Martha L Tena Suck, Juan C Balcázar-Padrón, Juan P Navarro-Garcia Llano, Alma Ortíz-Plata, Juan Luis Gómez-Amador

**Affiliations:** 1 Neuropathology, Instituto Nacional de Neurología y Neurocirugía Manuel Velasco Suárez, Mexico City, MEX; 2 Neurological Surgery, Instituto Nacional de Neurología y Neurocirugía Manuel Velasco Suárez, Mexico City, MEX

**Keywords:** cavernous malformation, collision tumor, brain calcified tumors, calcified cavernoma, pituitary adenoma

## Abstract

Collision tumors are rare neoplasms composed of two different types of histological tissues in the same organ. The most frequent association with cerebral cavernous malformations (CCMs) are meningiomas, gliomas, and gangliogliomas, while the most frequent sellar collision is between pituitary adenoma (PA) and craniopharyngiomas, and still very few cases have been reported. We present the case of a 43-year-old woman who started two months ago with a fall from her height followed by severe headache and bilateral hemianopsia. An isointense, enhancing sellar tumor, and a right frontal lesion compatible with CCM were observed on MRI. Surgery was performed through anterior interhemispheric and endoscopic transnasal approaches for the cavernoma and the sellar tumor, respectively, removing both lesions and sending them to pathology. The sellar tumor corresponded to a PA showing positive immunohistochemistry for prolactin and follicle-stimulating hormone (FSH). In the post-op period, the patient developed a seizure and diabetes insipidus, for which she received appropriate treatment. Our findings were conclusive with a collision tumor, since both lesions presented two different histological tissues. Different densities were observed in both lesions using imaging studies, which were later confirmed with histopathology and immunohistochemistry.

## Introduction

A true collision tumor is a rare entity composed of two histologically distinct neoplasms coinciding in the same organ, with different tumorigenic pathways and no histological admixture or intermediate cell population zone [1,2]. Cavernomas are vascular malformations characterized by closely packed vessels and abnormal capillaries surrounded by hemosiderin and gliosis, without the interposition of brain parenchyma [3]. The most common neoplasms associated with CCMs are meningiomas, gliomas, and gangliogliomas [4]. A pituitary adenoma (PA) associated with a calcified cerebral cavernous malformation (CCM) is extremely rare and difficult to diagnose preoperatively; pathological identification of the dual components is often the only way to ensure a final diagnosis [5].

We present the case of a rare association between a pituitary macroadenoma and a frontal partially calcified CCM in a young woman. We look to reinforce the hypothesis of both cavernomas and sellar neoplasms colliding as an entirely distinct entity.

## Case presentation

We present the case of a 43-year-old woman G3 P3 A0 C0 with menarche at age 13 and early menopause at age 26. One of her daughters suffered a cerebral hemorrhage, probably due to a CCM. She started two months ago with a fall from her height followed by severe headache and bilateral hemianopsia, for which she came to our emergency department.

**Figure 1:**
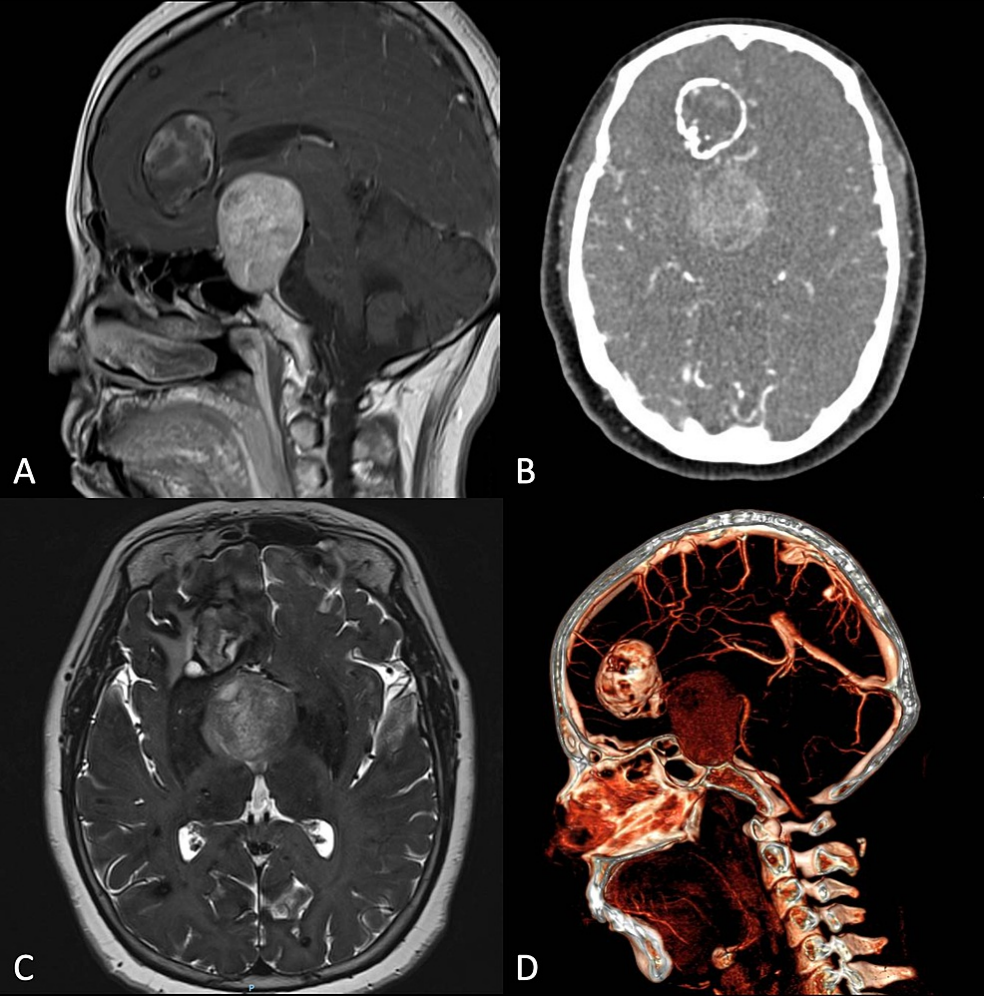
Brain MRI, CT scan, and CT angiography 3D reconstruction. A) Sagittal T2-flair brain MRI showing the sellar tumor with heterogenous intensities causing elevation of the third ventricle’s anterior floor and a calcified lesion on the frontal lobe with a hypointense core. B) Brain CT scan showing two lesions. A calcified lesion located at the right frontal lobe and a homogeneous lesion (PAs) at the sellar level. C) Axial T2-weighted brain MRI showing isointense sellar and right frontal heterogeneous lesions. D) CT angiography 3D reconstruction showing hypervascularization of both lesions.

On MRI, we observed an isointense, enhanced with contrast sellar tumor (36 x 42 x 40 mm), and a right frontal lesion compatible with CCM. The sellar tumor caused increased dimensions in this region by eroding the back of the sellar chair, thus producing elevation of the third ventricle's anterior floor (Figure 1A). In the right frontal lobe, it is possible to identify a space-occupying lesion (30 x 31 mm), calcified in the periphery, with a hypointense core shown on axial T1-weighted MRI and heterogenous on T2, consistent with a CCM (Figures 1B-1C). We also did a CT angiography 3D reconstruction, observing hypervascularization of both lesions (Figure 1D). It is also important to note that multiple other cavernous lesions appear in imaging studies, confirming the diagnosis of familial multiple cavernous malformation syndrome.

Surgery was performed through anterior interhemispheric (CCM) and endoscopic transnasal approaches (sellar tumor) to remove both lesions in the same surgical time, using a neuronavigation system to avoid injuring critical vascular structures. Both lesions were immediately sent to pathology after resection.

**Figure 2:**
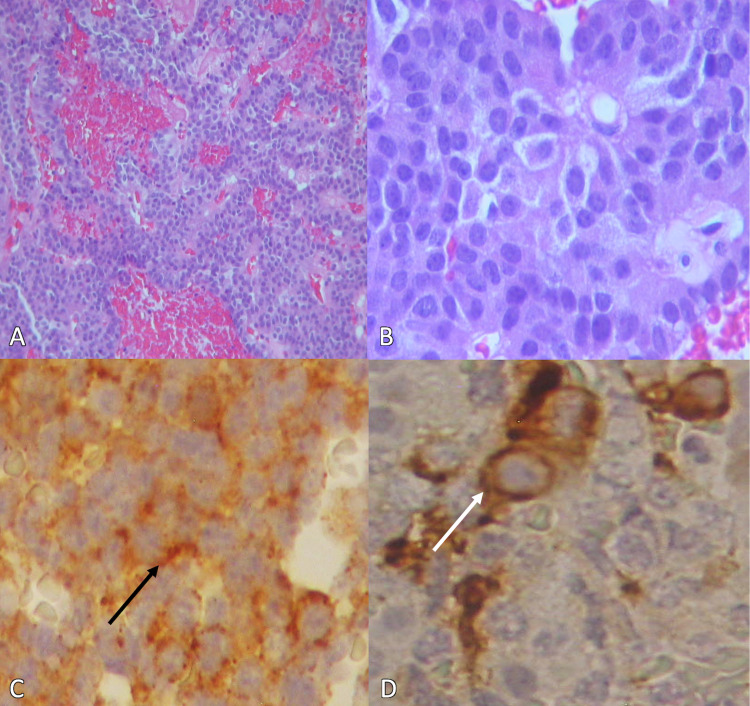
Pituitary adenoma histopathology report. A) Basophilic cells, congestive areas, and multiple necrotic foci (H&E x200). B) Pseudorossete formation (H&E x400). C) Immunohistochemistry showing positive prolactin expression (black arrow) (H&E x400). D) Immunohistochemistry showing positive FSH expression (white arrow) (H&E x400).

The sellar lesion corresponded to a neuroendocrine PA with a 0.46 g weight and a basophilic nodular cytoarchitecture that showed a soft consistency, smooth light brown surface, and scarce pseudorosettes formation with congestive vessels (Figures 2A-2B). The PA showed immunohistochemically positive prolactin and follicle-stimulating hormone (FSH) (Figures 2C-2D). Also, 3% ki67 labeling index and p53 weak positive immunoreaction.

**Figure 3:**
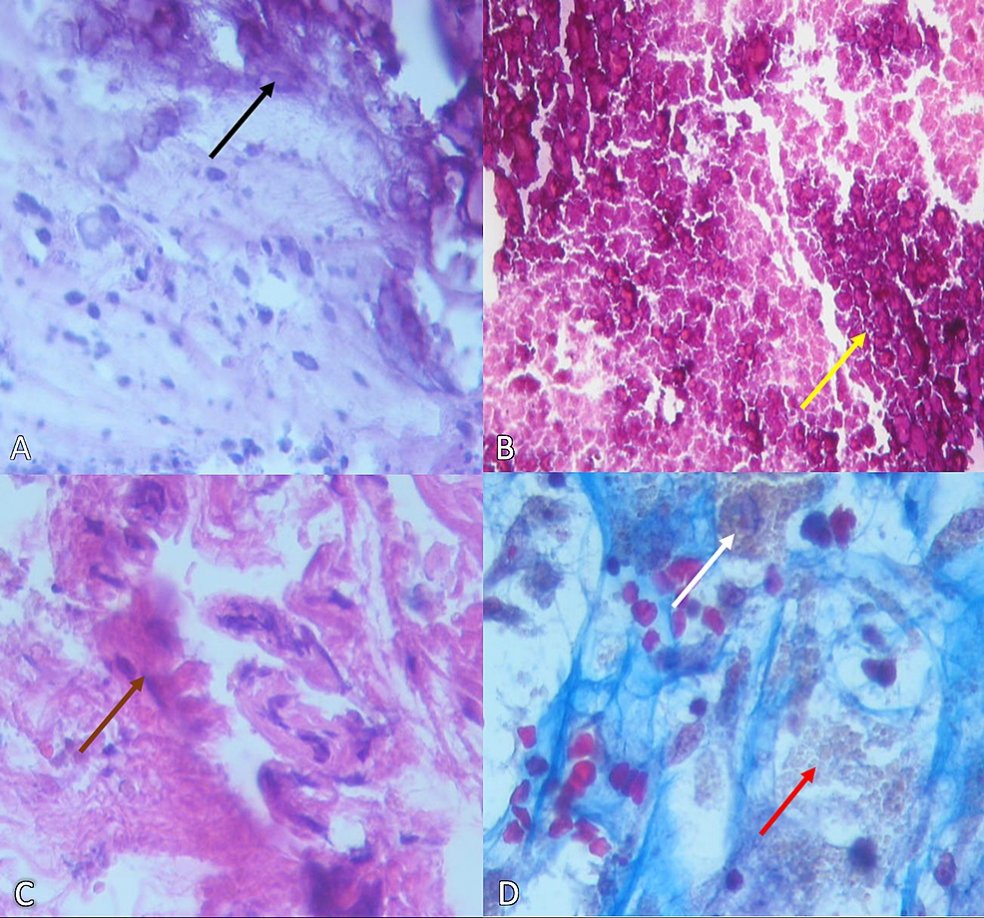
Cerebral cavernous malformation histopathology report. A) Capsule showing fibroconnective tissue with numerous dystrophic calcifications (black arrow) (H&E x200). B) Calcified dense eosinophilic areas (yellow arrow) (H&E x200). C) Hyalinized vessels with calcified walls (brown arrow) (H&E x400). D) Masson’s trichrome stain showing hemosiderophages (white arrow), and hemosiderin deposits (red arrow) (H&E x400).

The frontal lesion's capsule fragments weighed all together 1.84 g, composed of immersed small clots with a few brittle yellow laminar calcifications (Figure 3A). The content of the frontal CCM weighted 9.76 g, and it was described as irregular laminar appearance with abundant deposition of granular material, dystrophic calcifications, hemosiderin, and fibrous tissue compatible with vascular walls (Figures 3B-3D).

In the postoperative period, the patient developed an episode of generalized tonic-clonic seizure and diabetes insipidus due to cortical irritation secondary to manipulation of the surgical field for which she received appropriate treatment. 

Three months later, there was still PA's remnant on her follow-up MRI, deciding to initiate radiotherapy protocol. 

## Discussion

PA is a relatively common benign tumor (1 to 8 cases per 100,000 population) rising from the anterior hypophysis that constitutes 10%-15% of all intracranial tumors, representing 90% of sellar lesions, having their highest incidence in the third and fourth decades of life [1].

PAs clinically manifest by mass effect on neighboring structures due to tumor size and whether they are functional (the cell of origin will be responsible for the affected hormone) or non-functional. Classifying these tumors has been a complex task over the years [2,6]. In our case, despite being a pituitary macroadenoma (which is rare to be functional), the presence of early menopause and positive immunohistochemical staining for prolactin makes us think that this tumor could be a functional macroprolactinoma. However, we do not have a preoperative hormonal assessment to confirm its functionality.

When cavernoma calcified, the histological differential diagnosis is difficult, especially when what predominates are dystrophic calcifications with occasional blood vessels; however, the presence of abnormal focal vessels suggests CCM diagnosis [5].

These lesions may arise de novo or after radiation and may be associated with amyloid deposition [1,3]. CCMs can also follow an autosomal dominant inheritance pattern due to loss of function mutations in one of the three genes known as CCM1/KRIT1, CCM2/malcavernin, or CCM3/PDCD and can present anywhere in the central nervous system (CNS) [5]. 

Collision tumors are a rare entity composed of two histologically distinct neoplasms coinciding in the same organ. The most frequent sellar collision tumor is the association between PA and craniopharyngioma, and still, very few cases of this rare duality have been reported [7].

Since the CCM was entirely calcified, there is still a possibility that our diagnosis is wrong and could also correspond to a calcifying pseudoneoplasm of the neuraxis (CAPNON), calcified hemangioma, or meningioma. Finding thickened vessels could be a companion or a distractor; nevertheless, hemosiderin is the most crucial diagnostic factor for diagnosing CCM.

CAPNON is a rare fibro-osseous lesion that can develop anywhere in the neuroaxis, with a challenging diagnosis. Most cases have been diagnosed after surgical resection, and there have been reports exposing them as collision lesions, especially with lipoma [8].

To our knowledge, this is the first case reporting the association of a calcified CCM and a pituitary adenoma in a collision tumor. The scarce literature on this association could suggest that this is a mere coincidence; however, there is also room to think that the changes generated by one of the lesions could be fundamental in the pathogenesis of the other.

## Conclusions

Our findings may reinforce the hypothesis that the association between a CCM and sellar neoplasms may be a distinct pathological entity. The cavernoma calcification was probably caused by microhemorrhages or due to metabolic changes occurring as a consequence of the PA pathogenesis. As we previously stated, this work represents the first case reporting these findings. We encourage colleagues to contribute to these rare cases to better understand this pathological association in the future.
